# Folate Receptor-targeted Bioflavonoid Genistein-loaded Chitosan Nanoparticles for Enhanced Anticancer Effect in Cervical Cancers

**DOI:** 10.1186/s11671-017-2253-z

**Published:** 2017-08-29

**Authors:** Limei Cai, Rufen Yu, Xi Hao, Xiangcui Ding

**Affiliations:** Department of Gynaecology and Obstetrics, Ruian People’s Hospital, Ruian, Zhejiang 325200 China

**Keywords:** Genistein, Cervical cancer, Nanoparticles, Folic acid, Apoptosis

## Abstract

In this study, novel folic acid-conjugated chitosan nanoparticle was formulated for specific delivery of bioflavonoid, Genistein (GEN), to the cervical cancer cells. The prepared GEN-loaded chitosan nanoparticles (GCN) and folic acid-conjugated GCN (FGCN) showed smaller size with a controlled drug release profile. FGCN exhibited enhanced internalization potential in HeLa cells than that of GCN. The specific internalization of FGCN was mainly due to the affinity of folic acid (FA) with FRs-α which is present in large numbers in HeLa cells. The results revealed that FGCN has a specific affinity towards HeLa cells that will contribute to the better treatment. Folic acid-tagged nanoformulations exhibited a superior cytotoxic effect compared to that of non-targeted formulations. Consistently, IC50 value of GEN decreased from 33.8 to 14.6 μg/ml when treated with FGCN after 24 h incubation. The apoptosis studies indicated that the FGCN nanoparticles were then either GCN or free GEN in terms of anticancer activity. Overall, results revealed that folate conjugation to the delivery system might have great effect on the survival of cervical cancers that will be beneficial for overall cancer treatment.

## Background

Human Cervical cancer is one of the popular cancers in women’s reproductive age worldwide which is mainly resulted from human papillomavirus (HPV) [1]. The “International Agency for Research on Cancer” has cited reduced immune defense, smoking, and irregular sexual activity as main causes behind cervical cancers [2]. The latest advancement in medical technologies and diagnosis has a great impact on reducing the death rate in cervical cancers; nevertheless, this class of cancer is still increasing in developing nations such as China. Two prophylactic HPV vaccines (Gardasil and Cervarix) have been marketed for the treatment of cervical cancers; however, it showed effectiveness in adult patients only and failed to cheer in overall patients [3, 4]. Therefore, treatment options such as chemotherapy, surgery, and radiotherapy are regularly used; however, none is effective in treating cervical cancers. Chemotherapeutic agents or other small molecules have been reported to improve the cancer treatment efficiency if delivered specifically [5].

Recently, natural compounds have gained significant attention in the treatment of cancer as it is known to have less toxicity compared to that of chemotherapeutic drugs [6]. Especially, flavonoids present in many plants have reported to possess anti-inflammatory and anticancer properties. Genistein (4′,5,7-trihydroxyisoflavone) (GEN) is a potentially effective soy isoflavone that has received increased attention owing to its potent anticancer property [7, 8]. Studies have revealed that GEN inhibits protein tyrosine kinases and NF-κB and arrest the cell cycle at G2/M phase. This will lead to the downregulation of genes associated with the cell proliferation and cancer cell growth [9, 10]. The G2/M phase arrest will suppress the cancer cell migration, invasion, and induce cell apoptosis and cell death [11]. However, clinical potential of GEN was hindered due to its limited solubility (~1.45 μg/ml) and limited bioavailability [12]. Therefore, there is an immediate need to improve the physicochemical properties of GEN and its anticancer properties.

The application of nanotechnology in medicine is gaining increasing attention due to its ability to improve the anticancer properties of various small molecules [13]. The encapsulation of drug in a nanoparticle offers numerous advantages such as high stability, enhanced drug loading, higher internalization, favorable biodistribution, and improved pharmacokinetics [14]. Importantly, nanoparticles increase the anticancer effect of encapsulated compound by preferential accumulation in tumor tissues. Also, nanoparticles could reverse the multi-drug resistance (MDR) of tumor cells [15, 16]. In the present study, we have employed natural derived chitosan nanoparticles owing to its excellent biodegradability and biocompatibility profile. Moreover, primary amine group of chitosan offers some important properties including aqueous solubility and hemocompatibility. Also, amine group could be employed to change the surface of nanoparticles [17]. In order to increase the target specificity, we have conjugated folic acid on the surface of chitosan nanoparticles. Folic acid was selected as a targeting ligand owing to its overexpression in cervical cancer cells. The folate receptors are present in high or large numbers in cervical cancer cells. To be specific, FRs-α is present in normal cells also but is not exposed to blood circulation whereas it is highly exposed to blood circulation in case of cancer cells making it as an attractive target for folate-conjugated nanoparticles which might be internalized via endocytosis mechanisms [18, 19].

In the present study, we primarily aimed at increasing the anticancer property of GEN towards FR-α-overexpressed HeLa cervical cancer cells by encapsulating in FA-conjugated chitosan nanoparticles. The nanoparticles were characterized in terms of particle size, shape, and in vitro release kinetics. The targeting effect of FA was studied by cellular uptake assay in HeLa cancer cells. The anticancer effect of GEN and GEN-loaded nanoparticles were studied by cytotoxicity assay, live/dead assay, and apoptosis analysis.

## Methods

### Materials

Genistein was purchased from Aladdin Chemicals (Shanghai, People’s Republic of China). Folic acid, chitosan (85% deacetylated), was purchased from Sigma-Aldrich, China. EDC and NHS were also purchased from Sigma-Aldrich, China. All other chemicals are of reagent grade and used without any modifications.

### Preparation of GEN-loaded Folic Acid-conjugated Chitosan Nanoparticles

Prior to preparing nanoparticles, folic acid chitosan conjugate was prepared. Briefly, 44.1 mg of folic acid was dissolved in DMSO along with a mixture of 1-(3-dimethylaminopropyl)-3-ethyl carbodiimide hydrochloride (EDAC.HCl) and N-hydroxy succinimide (NHS) (molar ratio 1:1.5:1.5). The mixture was stirred for 30 min to allow activation of functional group. The organic solution was added drop-wise into chitosan solution (0.5% w/w) under continuous stirring. The stirring was continued overnight under dark conditions. The pH was made up to pH 8 by the virtue of adding 1 M sodium hydroxide. At the end, the mixture was centrifuged to separate yellow precipitate and washed with sodium carbonate and then dialyzed again with water.

To prepare nanoparticle, GEN, chitosan and chitosan-FA conjugate (10:1) were dissolved in DMSO. The DMSO solution was then added drop-wise into 0.5% Tween 80 solution under continuous stirring. The mixture was stirred for 3 h, and after all solvents evaporated, suspension was spun down for 30 min at 10,000 rpm at 4 °C. The supernatant was removed and evaluated by HPLC method. The samples were quantified by HPLC (LC 1100, Agilent Technologies, Santa Clara, CA, USA) using a reverse-phase C-18 column at 25 °C. A mobile phase of methanol/water (60/40, *v*/v) was used as the mobile phase at a flow rate of 1 ml/min.

$$ \mathrm{DL}\%=\mathrm{GEN}\ \mathrm{amount}\  \mathrm{in}\ \mathrm{NP}/\mathrm{Mass}\  \mathrm{of}\ \mathrm{NP}\times 100\% $$

$$ \mathrm{EE}\%=\mathrm{GEN}\ \mathrm{amount}\  \mathrm{in}\ \mathrm{NP}/\mathrm{Mass}\  \mathrm{of}\ \mathrm{GEN}\times 100\% $$

### Particle Size and Surface Morphology Analysis

The particle size and size distribution of nanoparticles were observed by Malvern Mastersizer 2000 (Zetasizer Nano ZS90, Malvern Instruments Ltd., UK). The samples were suitably diluted prior to analysis. All measurements were performed in triplicate.

The surface morphology of nanoparticle was evaluated by transmission electron microscopy (TEM, JEM-1230, JEOL, Tokyo, Japan). Briefly, nanoparticle dispersion was mixed with 2% PTX and put on the TEM grid and dried for 10 min under low infrared radiation. The samples were counter-stained with phosphotungistic acid (2%) and observed under TEM at an acceleration voltage of 100 kV.

### In Vitro Drug Release Study

The drug release study was performed by dialysis method. Briefly, 5 mg equivalent of GEN nanoparticles were suspended in 1 ml of release buffer (PBS, pH 7.4) and transferred to a dialysis tube (MWCO: 3500). The dialysis tube was placed in a Falcon tube containing the 25 ml of release buffer. The Falcon tube was placed in a shaker with gentle stirring at 37 °C. At pre-designated intervals, specific volume of sample was taken and replaced with fresh buffer or media. The drug released from the release medium was studied by HPLC.

### Cell Culture

Human cervical carcinoma cell line (HeLa cell) was obtained from ATCC, MS, USA and grown in DMEM containing 10% FBS and antibiotic mixture at 37 °C in a humidified atmosphere containing 5% carbon dioxide.

### Cellular Uptake

The targeting efficiency of nanoparticles (GCN and FGCN) was studied by cellular uptake analysis. In this study, fluorescent probe employed was Coumarin-6. The cells were placed in a 96-well plate and allowed to attach for 24 h. The culture medium was removed and replaced with fresh media containing GCN and FGCN and incubated for different intervals. The cells were then washed with PBS, lysed (Triton-X), centrifuged, and supernatant was collected. The supernatant was used to evaluate the fluorescence intensity by a microplate reader (GENios, Tecan, Switzerland). The absorption was measured at an excitation wavelength of 430 nm and emission wavelength of 485 nm.

### Cellular Uptake Imaging

Confocal laser scanning microscope (Olympus Fluoview FV-1000, Tokyo, Japan) was used to determine the qualitative cellular uptake in cancer cells. 2 × 10^5^ cells were seeded in a 6-well plate and incubated for 24 h. The media was removed and replaced with fresh media containing GCN and FGCN and incubated for 2 h. The cells were washed with PBS, fixed, and washed again. The cells were then observed using CLSM.

### Cytotoxicity Assay

The cell killing efficiency of free GEN, GCN, and FGCN was studied by means of MTT assay protocol. HeLa cells at a seeding density of 1 × 10^4^ cells were placed in a 96-well plate and allowed to attach for 24 h. The next day, cells were treated with free GEN, GCN, and FGCN in 100 μl of fresh media and incubated for 24 h. Afterwards, media was removed and washed twice with PBS. Ten microliters of DMEM containing MTT (5 mg/ml) was placed in a 96-well plate and allowed to stay aside for 4 h. The MTT solution was siphoned off and DMSO was added to dissolve formazan crystals which are corresponding to living cells. The absorbance of formazan was studied at 570 nm wavelength using a plate reader (Bio-Tek Instruments Inc., USA). The IC50 was also calculated using GraphPad Prizm software (version 17).

### Live/Dead Assay

HeLa cells at a seeding density of 2 × 10^5^ cells were seeded in a 6-well plate and incubated for 24 h. Next day, cells were treated with free GEN, GCN, and FGCN in 100 μl of fresh media and incubated for 24 h. Afterwards, media was removed and washed twice with PBS. The cells were processed according to the instruction given by the manufacturer (Molecular Probes, USA). The calcein AM and ethidium homodimer are respective live and dead cell dyes which are used for staining.

### Statistical Analysis

Data were statistically analyzed using *t* test. A *P* value less than 0.05 was used to show statistical significance. All of the experiments were performed in triplicate.

## Results and Discussion

**Fig. 1:**
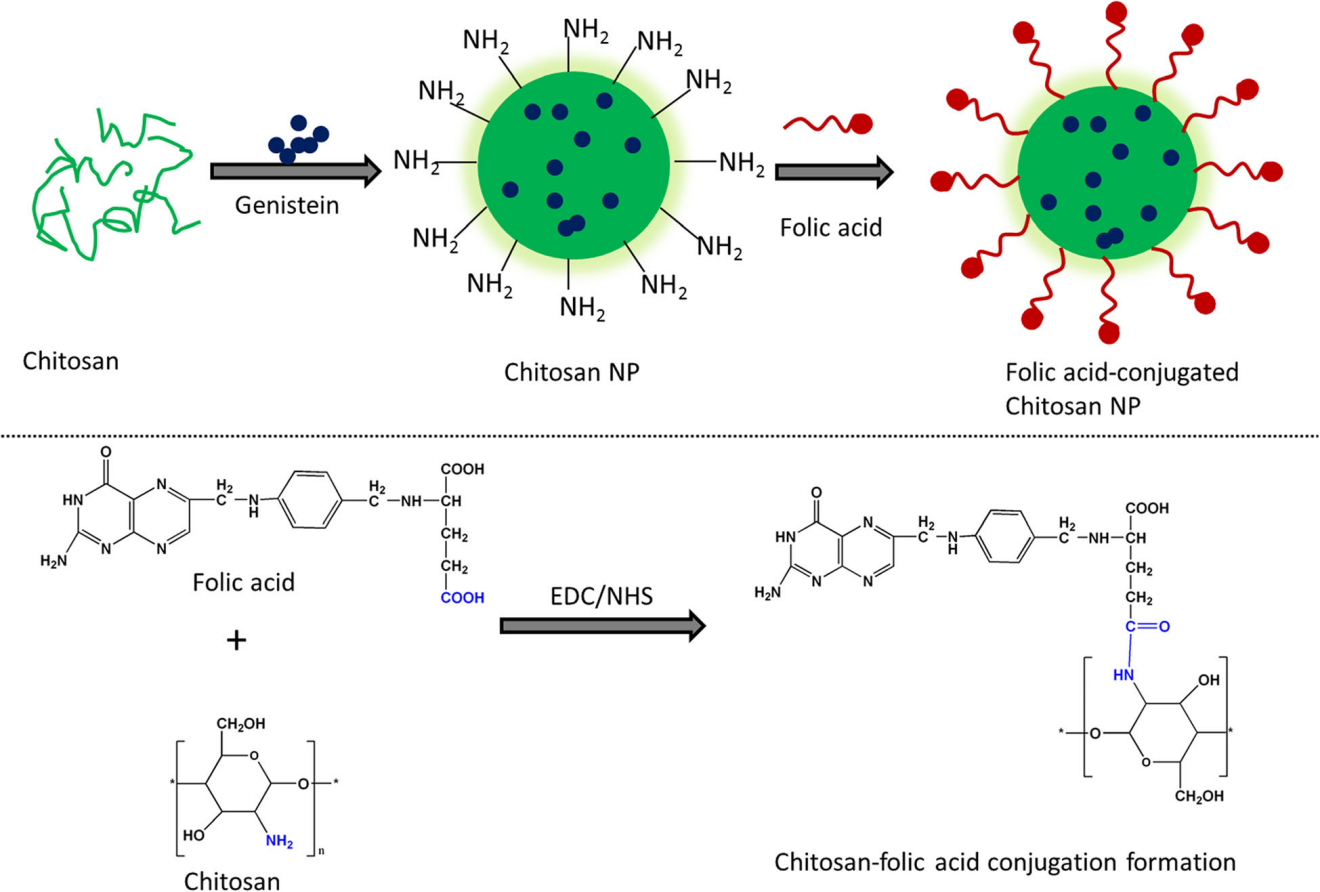
Schematic illustration of preparation of Genistein-loaded folic acid-conjugated chitosan nanoparticles

Human Cervical cancer is one of the popular cancers in women’s reproductive age worldwide which is mainly resulted from human papillomavirus. Therefore, treatment options such as chemotherapy, surgery, and radiotherapy are regularly used; however, none is effective in treating cervical cancers. Chemotherapeutic agents or other small molecules have been reported to improve the cancer treatment efficiency if delivered specifically. Recently, natural compounds have gained significant attention in the treatment of cancer as it is known to have less toxicity compared to that of chemotherapeutic drugs. Genistein has received increased attention owing to its potent anticancer property. However, clinical potential of GEN was hindered due to its poor solubility (~1.43 μg/ml) and low bioavailability. In the present study, we primarily aimed at increasing the anticancer property of GEN towards FR-α overexpressed HeLa cervical cancer cells by encapsulating in FA-conjugated chitosan nanoparticles (Fig. 1).

### Physicochemical Characterization of GEN-loaded Nanoparticle System

**Fig. 2:**
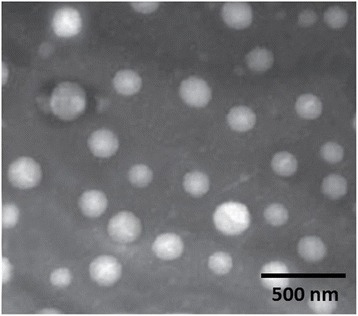
Transmission electron microscope of FGCN

**Fig. 3:**
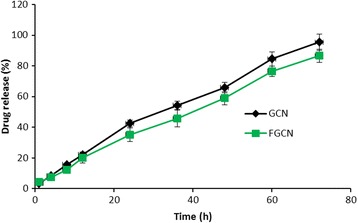
In vitro release profile of GCN and FGCN in phosphate buffered saline (pH 7.4). The amount of drug released was quantified by HPLC method, and the experiment was performed in triplicate

The size and zeta potential of particle system plays a crucial role in cellular internalization and systemic performance of anticancer drugs. Dynamic light scattering (DLS) was used to determine the particle size and size distribution of the GEN-loaded nanoparticles (Fig. 2). The average particle size of GCN was ~140 nm while FGCN was ~165 nm with excellent polydispersity index. The average hydrodynamic diameter of the FGCN is less than 200 nm, which is sufficient to penetrate the tumor tissues using enhanced permeation and retention effects. The small size of particles could resist the rapid clearance property of nanoparticles from the body (RES) [20]. The surface charge is considered a key indicator of the particle stability in suspension form. The average zeta potential of GCN was +26 mV while FGCN was +21.5 mV. A slight decrease in surface charge might be attributed to the substitution of amine group of chitosan. Moreover, GCN and FGCN showed a high entrapment efficiency of more than >95% indicating its suitability for systemic applications. The particle size and zeta potential of FGCN remained unchanged during storage for 3 months indicating its high storage stability (Fig. 3).

### Surface Morphology Analysis

**Fig. 4:**
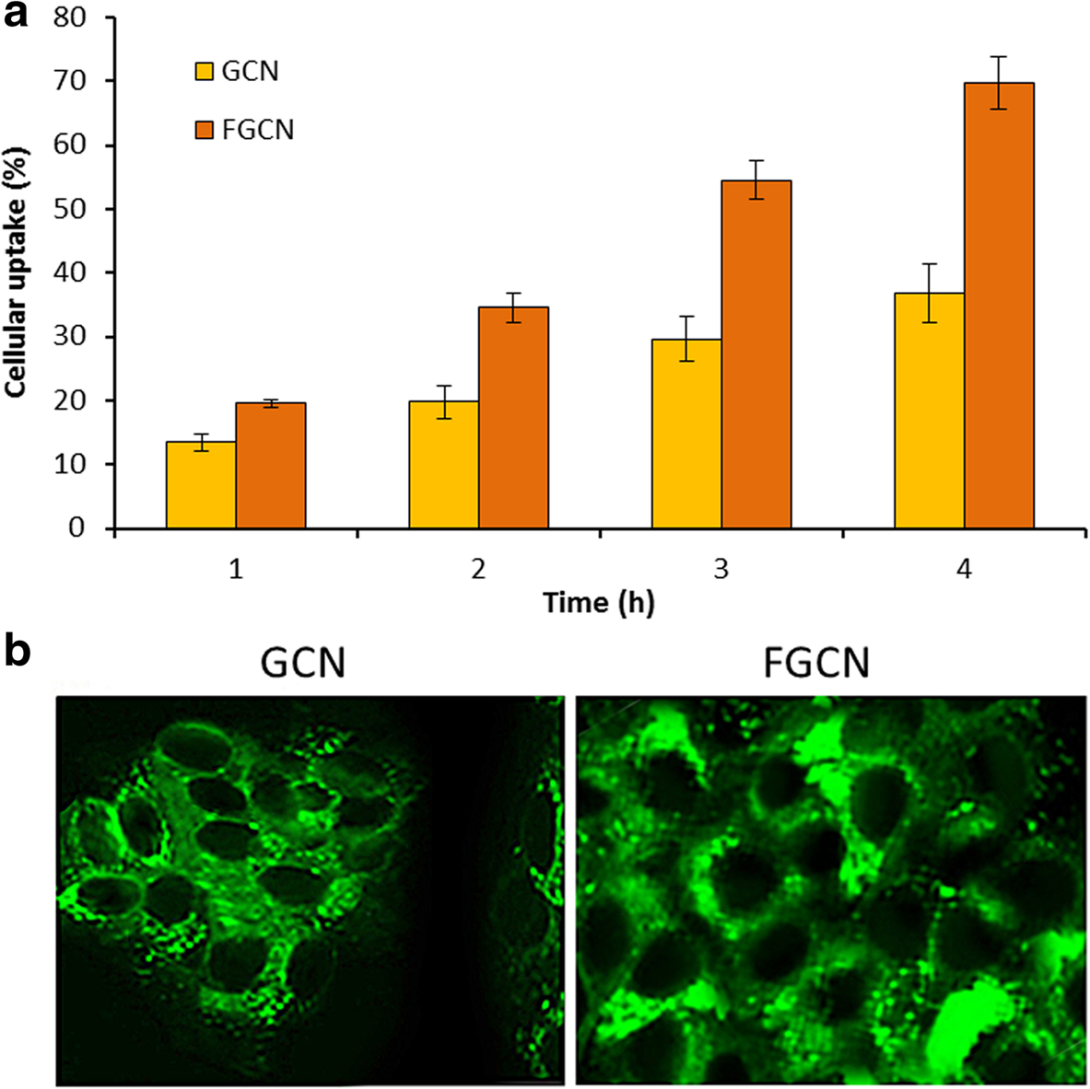
Cellular uptake analysis of GCN and FGCN in HeLa Cervical cancer cells. **a** Quantitative analysis of cellular uptake of nanoparticles by fluorescence method. **b** Confocal laser scanning microscope (CLSM)-based cellular uptake analysis. Coumarin-6 was used as a fluorescent probe

The surface morphology of optimized FGCN was characterized by TEM. The nanoparticles exhibited spherical shaped morphology and uniformly distributed in the copper grid. The particle size observed from TEM analysis was consistent with the DLS observation (Fig. 4).

### In Vitro Drug Release Kinetics

The release of GEN from GCN and FGCN was carried out using dialysis method. PBS was used to carry out the release study to simulate physiological conditions. As expected, release profile of GCN and FGCN were almost similar. Both the formulations exhibited a controlled release profile for GEN and exhibited monophasic release kinetics. Approximately 35–40% of drug was released from both the nanoparticle system at the end of 24 h. The trend of GEN release rate continued until the end of study period, and finally, ~90% of drug was released. The folate conjugation on the surface influences the low release of drug indicating the influence of presence of conjugated material. It has been reported that a controlled release of drug from NP was attributed to the lengthening of path length of drug to the outer release media. Overall, a controlled release of drug in pH 7.4 conditions suggests that much of the drug will be intact and will accumulate in the tumor tissues with time.

### In Vitro Cellular Uptake Study

The intracellular concentration of GEN is very important to elicit its cytotoxic action in the cancer cells. Therefore, cellular uptake potential of NP system is very important from a therapeutic efficacy perspective. The coumarin-6-loaded GCN and FGCN were incubated to cancer cells for different time points. Both the NP systems exhibited a time-dependent cellular uptake with maximum cellular uptake at 4 h. As expected, folic acid receptor-targeted FGCN exhibited a statistically (*p* < 0.05) higher cellular uptake in HeLa cancer cells. Once again, predominant uptake of FGCN in most cases will be due to the availability of FA on the particle surface that might facilitate the interaction of ligand with the corresponding receptors and which is present in high numbers in HeLa cells [21].

The cellular uptake is further confirmed by microscopic imaging. The cells were incubated with respected formulations and incubated for 2 h. As seen, high fluorescent intensity was observed for FGCN exposed cancer cells compared to that of GCN-treated cells. These results indicated that folic acid-conjugated FGCN had a specific affinity for the cancerous, HeLa cells owing to ligand-receptor (FA-FR-α) recognition.

#### In Vitro Cytotoxicity

The in vitro cytotoxic activity of GEN, GCN, and FGCN on HeLa cancer cell was studied by MTT assay protocol. As seen, nanoformulations of GEN was more effective in killing the cancer cells compared to that of free GEN. All the formulations however exhibited a typical dose-dependent cytotoxic effect in the cancer cells. As expected, folic acid-tagged nanoformulations exhibited a statistically (*p* < 0.05) higher cytotoxic effect compared to that of non-targeted formulations. The superior anticancer effect of FGCN was attributed to the specific receptor-mediated nanoparticle internalization in the cancer cells that might increase the concentration of drug in the intracellular environment. Further, ability of nanoparticles to control the release of encapsulated compound also contributed to the high cytotoxicity of FGCN. The IC50 value was used to evaluate the real cytotoxic effect of formulations on the cancer cells. Generally, it is the concentration which is required to kill half of the original cells. The results showed that IC50 value of GEN decreased from 33.8 to 26.5 μg/ml when treated with GCN. Importantly, IC50 value decreased to 14.6 μg/ml when treated with FGCN after 24 h incubation. Nanoparticles have been reported to reduce the MDR effect and thereby expected to increase the anticancer efficacy of GEN by reducing its efflux from cells mediated by the P-glycoprotein. The superior anticancer effect of FGCN was consistent with its high cellular uptake in HeLa cancer cells [22].

#### Microscopic Analysis

**Fig. 5:**
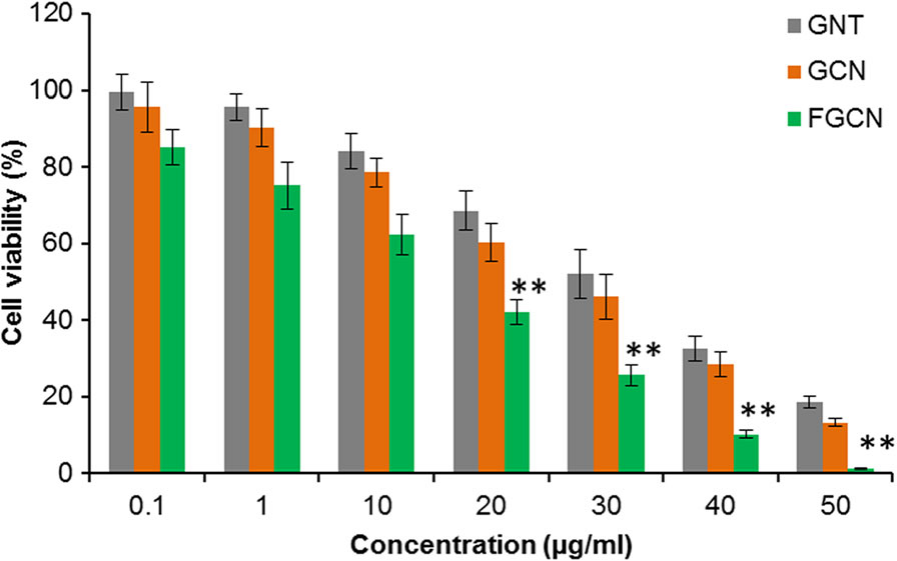
In vitro cytotoxicity analysis of GEN, GCN, and FGCN in HeLa cancer cells. The cells were treated with the respective formulations and incubated for 24 h. **p < 0.01 is the statistical difference between FGCN and GEN.A

Morphological analysis of cancer cells were carried out after treatment with anticancer drugs. The formulations were exposed to cancer cells and incubated for 24 h. The control cells were normal and spread on the cover slip of the well plate whereas cells shrank in the formulations-treated group. Consistent with cytotoxicity assay, morphology of cancer cells were different for different formulations. As shown, FGCN-treated cells were fewer in number and round morphology indicating the higher cytotoxicity of the formulations. The GCN also induced remarkable changes in the cancer cells owing to its reasonably higher uptake in cancer cells. Results clearly reveal that chitosan-coated nanoparticles will be beneficial for the cervical cancer treatments (Fig. 5).

#### Live/Dead Assay

**Fig. 6:**
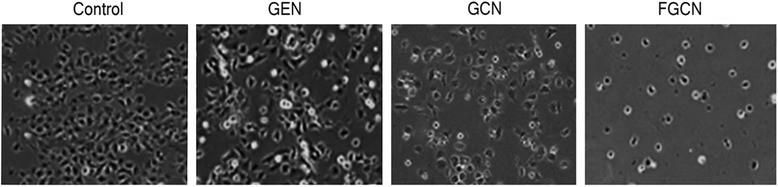
Morphological analysis of HeLa cancer cells after treatment with GEN, GCN, and FGCN

The cytotoxic potential of individual formulations was further studied by live/dead assay. The amount of cells alive and dead after the drug treatment (for 24 h) was visualized from green fluorescence intensity. Calcein AM and ethidium bromide dye are representative live and dead cell markers which are applied to cells treated with formulations. The remaining live cells present after drug treatment takes up calcein and emit green fluorescent light emission (488 nm). As shown, untreated cells showed high fluorescence intensity (green fluorescence), and treatment of free GEN also did not reduce the growing cells. On the other hand, FGCN showed fewer live cells and higher dead cells (low fluorescence intensity), indicating the potent effect. The high cell death rate of FGCN-treated group was attributed to the higher nanoparticle internalization in cancer cells. The result is consistent with the cytotoxicity assay (Fig. 6).

### Apoptosis Analysis

**Fig. 7:**
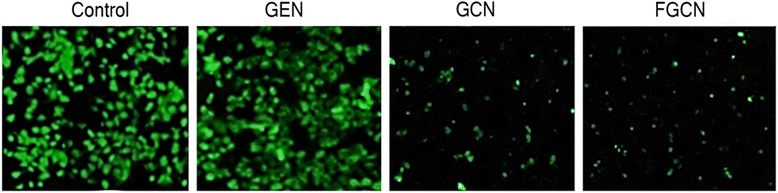
Live/dead assay of HeLa cancer cells after treatment with GEN, GCN, and FGCN. The green fluorescence indicates the live cells

**Fig. 8:**
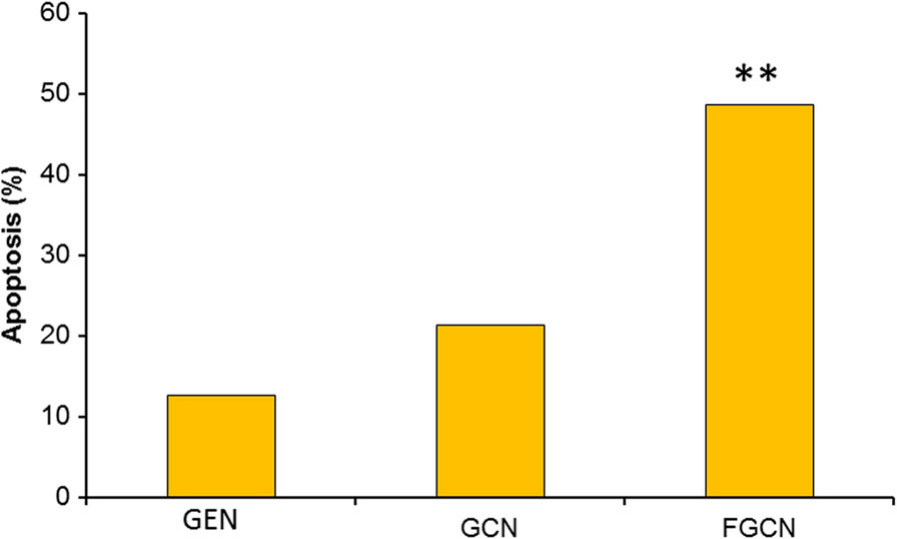
Apoptosis analysis of HeLa cancer cells after treatment with GEN, GCN, and FGCN. Flow cytometer was used to analysis the proportion of cell apoptosis. **p < 0.01 is the statistical difference between FGCN and GEN

In this study, we have designed a unique folic acid receptor-targeted delivery system that could increase the intracellular concentration of GEN and improve the anticancer effect in cervical cancer cells. Therefore, in order to analyze the apoptotic induction property of free GEN, GCN, and FGCN NP, cells were assessed by flow cytometry after annexin V/PI double staining. Figure 7 shows that cells treated with free GEN did not induce appreciable apoptosis of cancer cells while GCN (~22%) was effective in inducing the apoptosis in these cancer cells. As expected, FGCN exhibited a remarkable apoptosis of cancer cells (~55%) indicating its superior anticancer effect. The higher anticancer effect of formulations was due to the specific affinity of ligand to the corresponding receptors which in turn increased the drug concentration in the cancer cells. Results clearly reveal that chitosan-coated nanoparticles will be beneficial for the cervical cancer treatments. The nanotechnology platform for the anticancer drugs increased the toxicity in the cancer cells and increased the therapeutic efficacy of antitumor agents (Fig. 8).

## Conclusions

In this study, novel folic acid-conjugated chitosan nanoparticle was formulated for specific delivery of bioflavonoid, Genistein (GEN), to the cervical cancer cells. FGCN exhibited enhanced internalization potential in HeLa cells than that of GCN. The results revealed that FGCN has a specific affinity towards HeLa cells that will contribute to the better treatment. Folic acid-tagged nanoformulations exhibited a superior cytotoxic effect compared to that of non-targeted formulations. Consistently, IC50 value of GEN decreased from 33.8 to 14.6 μg/ml when treated with FGCN after 24 h incubation. The apoptosis studies indicated that the FGCN nanoparticles were either GCN or free GEN in terms of anticancer activity. Overall, results revealed that folate conjugation to the delivery system might have great effect on the survival of cervical cancers that will be beneficial for overall cancer treatment. The study on clinical animal model wills the prospect the next step of the present study.

## Abbreviations

EPR: Enhanced permeation and retention
FGCN: folic acid-conjugated GEN-loaded chitosan nanoparticles
FR: Folate receptors
GCN: GEN-loaded chitosan nanoparticles
GEN: Genistein
